# Dietary Interventions for Type 2 Diabetes: How Millet Comes to Help

**DOI:** 10.3389/fpls.2016.01454

**Published:** 2016-09-27

**Authors:** Jason Kam, Swati Puranik, Rama Yadav, Hanna R. Manwaring, Sandra Pierre, Rakesh K. Srivastava, Rattan S. Yadav

**Affiliations:** ^1^Institute of Biological, Environmental and Rural Sciences, Aberystwyth University, Gogerddan, AberystwythUK; ^2^International Crops Research Institute for the Semi-Arid Tropics, PatancheruIndia

**Keywords:** millet, diabetes, hyperglycaemia, crop, nutritional characteristics, diet, plant breeding

## Abstract

Diabetes has become a highly problematic and increasingly prevalent disease world-wide. It has contributed toward 1.5 million deaths in 2012. Management techniques for diabetes prevention in high-risk as well as in affected individuals, beside medication, are mainly through changes in lifestyle and dietary regulation. Particularly, diet can have a great influence on life quality for those that suffer from, as well as those at risk of, diabetes. As such, considerations on nutritional aspects are required to be made to include in dietary intervention. This review aims to give an overview on the general consensus of current dietary and nutritional recommendation for diabetics. In light of such recommendation, the use of plant breeding, conventional as well as more recently developed molecular marker-based breeding and biofortification, are discussed in designing crops with desired characteristics. While there are various recommendations available, dietary choices are restricted by availability due to geo-, political-, or economical- considerations. This particularly holds true for countries such as India, where 65 million people (up from 50 million in 2010) are currently diabetic and their numbers are rising at an alarming rate. Millets are one of the most abundant crops grown in India as well as in Africa, providing a staple food source for many poorest of the poor communities in these countries. The potentials of millets as a dietary component to combat the increasing prevalence of global diabetes are highlighted in this review.

## Type 2 Diabetes Overview and Associated Complications

Diabetes is a chronic disease that is characterized by high level of blood glucose also known as hyperglycaemia. According to WHO 2015 published figure^1^, 9% of the world population aged 18 and above has contracted diabetes and an estimated 1.5 million deaths per year are attributed to diabetes directly. It is well known that glucose level of a diabetic patient increases dramatically beyond the normal range after a meal. It is also true that their blood glucose level would soon drop as the body failed to store the excess glucose for later use.

Diabetes is classified into Types 1 and 2. Type 1 diabetes is also known as juvenile diabetes or insulin-dependent diabetes as the patients’ pancreas cannot produce or produces little insulin and often presents itself from childhood (Diabetes.co.uk, 2016c). Type 2 diabetes (T2D), however, often first appears in adults when the body becomes resistant to insulin or fails to make sufficient amounts of insulin (Martin et al., 1992; Weyer et al., 2001). T2D comprises 90% of people with diabetes around the world (NHS choice, 2014). This can largely be the result of excess body weight and physical inactivity. Added complication to T2D is that it presents less marked symptoms than Type 1 diabetes and is often diagnosed only when complications have already arisen.

Major complications caused by hyperglycaemia include atherosclerosis that hardens and narrows the blood vessels. Other diabetes-related complications are heart disease, stroke, retinopathy, and kidney failure (Bitzur et al., 2009; Sone et al., 2011). Diabetic retinopathy leads to blindness by causing cumulative damage to the small blood vessels in the retina and contributes to 1% blindness globally. Similarly, kidney failure due to prolonged restricted blood flow is a very common complication. Elevated blood glucose can also cause nerve damage (Boulton et al., 2005) that may lead to the need of limb amputation (Brownlee, 2001). Such ailments reduce the patients’ quality of life, and potentially relationship with others around them.

Other additional complications also include increased bone fracture risks in both Types 1 and 2 diabetics (Saito et al., 2006; Vestergaard, 2006; Oei et al., 2015). Interestingly, however, Types 1 and 2 diabetics have lower and higher bone mineral density than healthy subjects, respectively, even though both are at elevated risk of fracturing (Ma et al., 2012; Oei et al., 2013; Strotmeyer, 2013; NIH Osteoporosis and Related Bone Disease National Resource Center, 2015). Fracturing risks associated with different bone mineral densities can be explained by other diabetes related factors. Firstly, T2D subjects are often found to have higher body mass index and less physical exercise making any fall carrying a higher risk of fracture (Vestergaard, 2006; Ma et al., 2012). Also, other complications such as retinopathy, as well as often-associated habits such as higher alcohol consumption interfering with the sense of balance, making fall more frequent adding to the fracture risk (Rubin, 2015). Physiologically, the rise of glucose level in the body interferes with glycation that subsequently reduces collagen cross-linking and results in more brittle bone despite the higher bone mineral density (Hein et al., 2006; Saito et al., 2006; Vestergaard, 2006). In addition, lower bone turnover rate causes poor fracture healing in diabetic patients, through interference from alternated glycaemia with a key bone remodeling regulator, the parathyroid hormone (Rubin, 2015). Therefore, in many converging ways, T2D contributes toward a higher fracture risk. Subsequently, fractures further restrict the mobility of patients, worsening the condition of diabetes.

Another symptom that a T2D patient may have to endure involves muscle fatigability due to poor glycaemic control (Halvatsiotis et al., 2002). This in turn causes tiredness and lack of energy often demotivate patients from engaging in physical exercises. Also, patients loose muscle mass as the body draws energy from breaking down muscles. Such abnormal anabolism of muscle makes muscle mass loss one of the many dangers that a diabetic patient have to face (Møller and Nair, 2008; Bassil and Gougeon, 2013). The subsequent loss of motor function adds further physical as well as psychological complications to the patients.

## Why Diet Is an Important Intervention

Diabetic patients experience fluctuation of blood glucose causing various health complications (Coppell et al., 2010; Jali et al., 2012). One of the interventions is to control this fluctuation using dietary regulation with or without exercise and medications. With the number of people suffering from diabetes on the rise globally (WHO, 2016), it is imperative to develop preventative measures involving intervention of diet and lifestyle, which would greatly reduce the risk of developing diabetes (Diabetes Prevention Program Research Group, 2002; Lindstrom et al., 2006). In addition, the risk of subsequent health complication can be reduced with the right treatment (Gaede et al., 1999). Indeed, decline of diabetes-related complication such as retinopathy has been shown to be positively correlated with earlier intervention (Vallance et al., 2008). This results in the reduction of financial burden on the health services as well as improving the well-being for the patients.

A study by the Diabetes Prevention Program Research Group (2002) concluded that lifestyle intervention resulted in 39% lower incidence of diabetes than another group using only metformin, an interventive medication for people who are at risk of diabetes. The same study found that both lifestyle intervention and metformin were effective in restoring normal fasting glucose values. Indeed, lifestyle intervention, including dietary habit reform, was found to be more effective in restoring normal glucose values after ingestion. Though this experiment was not designed to investigate dietary change or increased physical activities individually, it was undeniable that both contributed significantly toward lowering the risk of developing diabetes.

It is notable that there are different prescribed diets across continents and countries (Ajala et al., 2013). Based on extensive literature search, Ajala et al. (2013) concluded that lower carbohydrate and Mediterranean diet lowers HbA1c count (HbA1c is a test of blood glucose level over a period of time). In some cases, vegetarian (Kahleova et al., 2011) and low-glycemic index (GI) (Ben-Avraham et al., 2009) diets were recommended to help reduce the use of diabetic medication.

Interestingly, two studies comparing high-protein diet and high-carbohydrate diet showed no significant differences in weight loss between the two diet groups (Brinkworth et al., 2004; Larsen et al., 2011). In fact Larsen et al. (2011) showed that the two different diets did not produce any difference in the level of HbA1c. That may explain as to why there are variations in accepted dietary recommendations for T2D patients. However, Brinkworth et al. (2004) did observe better improvement on blood pressure for those that were on high protein diet and concluded that it may have a long term favorable effect on cardiovascular risk profile. Both papers noted that the two different diets improved the general health for T2D patient.

An interesting conclusion was drawn in a study with more than 37 000 participants, of which 915 incidences of diabetes were reported over 10 years (Sluijs et al., 2010). This study has confirmed that a positive correlation exists between higher GI food and diabetes and that fiber intake inversely correlated with diabetes. Interestingly, only starch in the carbohydrate sub-types was found to be related to diabetes risk. They concluded that diet constituents play a major part in controlling diabetes. These are just a few examples of how dietary intervention can improve diabetic condition.

## Current Recommended Diets for Diabetics

Diets play an important role in controlling the on-set of diabetes, as there is a positive correlation between dietary glycaemic load and increased diabetic risk (Sluijs, 2011; Greenwood et al., 2013). Eating the ‘wrong’ thing can accelerate the onset of diabetes. On the bright side, study has shown that dietitians can select appropriate intervention diets based on the client’s lifestyle (Franz et al., 2010). Such options reduce the requirement to impact on the patients’ lifestyle, thus increasing the likelihood of the treatment to be successful.

Problems with dietary intervention, however, also arise from different standards from different countries. For example, the United Kingdom has a very different standard than the rest of Europe (Ben-Avraham et al., 2009; Ajala et al., 2013). Also the American Diabetes Association (ADA) and the Canadian Diabetes Association (CDA) show differences among themselves, as well as with their European counterpart (Ben-Avraham et al., 2009). More differences can be observed when Japan, South Africa, and India are added into the collective. This may mainly be due to the lifestyle differences between different cultures, but also to different dietary requirements in their local climate. Also, there seems to be many variations in terms of the length of studies on dietary intervention on T2D (Salas-Salvado et al., 2011). These studies ranged from less than 4 and up to 23 years with variable dietary comparison as well as sample sizes. Further, some benefits from life style intervention requires longer observation period with higher sample size to draw a more accurate conclusion (The Look Ahead Research Group, 2010).

A quick scan on recent publications of various nutrition recommendations for diabetics is summarized in **Table 1** (Ben-Avraham et al., 2009; Salas-Salvado et al., 2011; Ajala et al., 2013). All these recommendations include carbohydrate, fiber, protein and fat. Most countries would recommend for carbohydrate intake to range from 40 to 60%, with the exception of India (>65%). Fat intake varies between different diets while protein intakes varied between 10 and 35%. Most recommendations would also include advice for fiber intake except the US. Yet the defined unit for fiber differed from one another. So there is a lack of uniform recommendation of diet globally. One, however, may question the sensibility of having a uniform recommendation as people from different regions have very different lifestyles as well as physiologies.

**Table 1:** Various recommendations for diabetic diet summarized in publications (Ben-Avraham et al., 2009; Salas-Salvado et al., 2011; Ajala et al., 2013).

Organizations	Carbohydrates	Fiber	Protein	Fat
American Diabetes Association (ADA)	50–60%	Not specified	10–15%	30–35%
The British Diabetic Association/Diabetes UK	45–60%	<30 g/day	30–35%	<35%
European Association for the Study of Diabetes (EASD)	45–60%	Increase fiber with low-GI food	10–20%	35%
The Canadian Diabetes Association (CDA)	50–60%	25–35 g/day	11%	30%
Japan	60%	1 fruit + 400 g vegetables	15–20%	20–25%
South Africa	50–60%	40 g/day	12–30%	≤30%
India	>65%	40 g/day	12–20%	<21%
Mediterranean diet	40–42%	~23 g/day	~16%	40–42%

Salas-Salvado et al. (2011) suggested a dietary regime of plant-based food with a lower intake of meat, sweets, high-fat dairy and refined grains, which is commonly known as Mediterranean diet, for lowering the risk of diabetes. This recommendation has attracted attention (American Diabetes Association, 2016; Diabetes.co.uk, 2016b) and research (Trichopoulou et al., 1995, 2003). Note that Mediterranean diet has a diversity of definition depending on geographical location. The Mediterranean diet here is referring to that of Greece and southern Italy in the early 1960s (Willett et al., 1995). Mediterranean diet is characterized by a high intake of vegetables, legumes, fruits, nuts, cereals, and a high intake of olive oil but a low intake of saturated lipids, a moderately high intake of fish (depending on the geographical location), a low-to-moderate intake of dairy products, a low intake of meat and poultry, and a regular but moderate intake of wine during meals. Total fat in this diet may be higher (40–42%, **Table 1**) but the mono-unsaturated:saturated fat ratio is above two. However, Salas-Salvado et al. (2011) admitted that this diet alone may not suffice in controlling incidence of diabetes.

The limited information in literature on the role of micro-nutrients seems to indicate that they can have significant influence on diabetes (O’Connell, 2001). The latter part of this review will attempt to investigate the relationship of micro-nutrients with health benefits against diabetes.

### Carbohydrates and Fiber

Starch is a carbohydrate that provides much needed energy for day-to-day activities. It is essentially composed of linear amylose involving α-1,4 linked _D_-glucopyranosyl units and branched amylopectin which is also interconnected by α-1,6 glycosidic linkages (Zhang et al., 2006a,b; Lehmann and Robin, 2007). The chain length and branching pattern, as well as the amylose to amylopectin ratio, all play a role in digestion efficiency (Zhang et al., 2006a,b).

Starch is generally divided into three different digestibility types: rapidly digestible starch (RDS), slowly digestible starch (SDS), and resistant starch (RS) (Englyst et al., 1992). RS is characterized by the fact that it is unable to be broken down in the small intestine and is therefore passed onto the large intestine. RS is further divided into three different types: physically inaccessible starch (RS_1_), resistant starch granules (RS_2_), and retrograded amylose (RS_3_) (Sajilata et al., 2006). RDS is referred to the starch fraction that is transformed into glucose soon upon ingestion (20 min). RDS is digested and absorbed in the duodenum and proximal regions of the small intestine (Englyst et al., 1992). The rapidly digestible nature of the RDS causes a rapid rise of blood glucose followed by a subsequent hypoglycaemia. On the other hand, SDS is referred to starch that breaks down into glucose over a longer duration (20–120 min; Lehmann and Robin, 2007; Zhang and Hamaker, 2009). The slow release of SDS improves overall blood glucose control as well as providing stable energy to patients with T2D.

While RS is not digested and absorbed by human as energy, it has a positive role against diabetes. Johnston et al. (2010) has discovered that consumption of resistant starch improves insulin sensitivity. It does not affect body weight, fat storage in muscle, liver or visceral depots significantly. Also, it helps managing meal-associated hyperglycaemia (Lehmann and Robin, 2007). This is particularly important for people who are at risk of, or suffering from, T2D.

Slavin (2005) defined dietary fiber as non-digestible carbohydrates and lignin that are derived from plant. It can be further classified into soluble and non-soluble fiber (Slavin, 2003). It has already been known for a while that fiber plays a positive role in glycaemic control (Jenkins et al., 1978; Chandalia et al., 2000). It is recommended that an increase of dietary fiber, particularly the soluble type, should be taken by T2D patients (Wood et al., 1990; Slavin, 2005). It is reasoned that soluble fiber reduces enzyme access to its substrates through viscosity effect. Slavin (2005) has concluded that fiber intake and obesity are inversely correlated, indicating that fiber is an important instrument in starch and cholesterol control.

Starch granule structure is organized in concentric layers with amylopectin as branching polymer and amylose as liner polymer (Gallant et al., 1992; Zhang et al., 2006a). These layers are organized in crystalline structures that form different patterns, resulting in variable enzymatic digestion susceptibility. Note that starch physical structure alone may not necessarily reflect glucose response in the gastrointestinal tract. For an example, physical mixture of starch and protein can influence digestion time (Zhang and Hamaker, 2009). Therefore, partially adding non-starch filler such as protein or fiber, potentially can produce a lower glycaemic response. Like many other nutrients, starch can be modified by other additives (Agama-Acevedo et al., 2012). By adding unripen banana flour into cookies, Agama-Acevedo et al. (2012) have reduced RDS and increased SDS contents. Hence, even if the intrinsic RDS, SDS, and RS ratios of a type of food may give indication of GI responses, it can be influenced by other nutrients present in the meal.

Another carbohydrate to consider is sugar intake. It can often provide rapid energy, in case of emergency of glucose depletion, for the patient (American Diabetes Association, 2015b; NHS choice, 2015). On the other hand, most sweet things (e.g., drinks/cakes/sweets etc.) which people enjoy often contain too much sugar, thus causing hyperglycaemia (Diabetes.co.uk, 2016a). Sugar-sweetened foods, particularly beverages taken in large quantity, are generally not recommended for anyone and particularly those who have or are at risk of diabetes. Although reducing sugar intake may seem to be a logical step to take in reducing blood sugar level, eliminating it completely from diet would be a mistake (American Diabetes Association, 2015a; Diabetes U. K., 2016). There are, however, alternatives to those who have a sweet tooth and find sweets irresistible. There are different sugar substitutes such as sucromalt which shows delayed glucose and insulin fall (Grysman et al., 2008). Another sugar substitute that is often used, fructose, has the ability to blunt glycaemic and insulin responses. The latter may seem to be advantageous on glycaemic control, but the corresponding physiological response on human may indicate otherwise (Havel, 2005). Problems arise as fructose is not controlled by the glucose homeostasis system and high consumption subsequently leads to dysregulation of energy homeostasis. This may result in hyperlipidaemia and obesity that further put strain on the person already suffering from diabetes (Elliott et al., 2002). When it comes to sugar, perhaps moderation would be the consensus between many dietary recommendations. A monitored diet maybe required due to sugar craving caused by previous excess consumption (Avena et al., 2008).

### Protein/amino acid (leucine)

Protein has been repeatedly identified as an important component for dietary strategies for diabetics (Ezeogu et al., 2008; Ben-Avraham et al., 2009; Singh et al., 2010; Ajala et al., 2013). There are many findings of proteins or specific amino acids such as leucine that have some positive influence on the condition of diabetic patients. These include improved glycaemic control and muscle loss prevention (Manders et al., 2006; Zhang et al., 2007; Melnik, 2012; Norton et al., 2012).

An advantage of having protein in the food matrix is that it can influence the rate of starch digestion (Singh et al., 2010). A study on *Sorghum* concluded that proteins with disulfide bonds interfere with starch digestibility (Wong et al., 2009). Such influence includes delayed starch digestion and control of postprandial hyperglycaemia. Another study on wheat starch-protein on glycaemic response and *in vitro* digestion has also concluded that the removal of protein such as gluten in wheat flour causes a rise of postprandial blood-glucose level (Jenkins et al., 1987). These evidences support the notion that protein integrated in the food matrix can improve postprandial glycaemia.

T2D shows a positive correlation with patients’ muscle mass loss (Bassil and Gougeon, 2013; Leenders et al., 2013). Therefore, muscle mass maintenance is part of the issue that needs to be considered. Since muscle is made up of protein, intake of amino acid is vital. In particular, the amino acid leucine has been known to induce muscle growth (Dardevet et al., 2000; Crozier et al., 2005; Katsanos et al., 2006). It has been found to be most effective through ingestion. In fact, much of the body building products such as protein shake contains leucine for muscle building as a significant ingredient (Hennessy, 2013). Protein supplement such as whey has a substantial level of leucine added to the product (Jakubowicz and Froy, 2013). It is often mentioned that consuming protein supplement such as whey has insulinotropic as well as glucose lowering, effects. This is why leucine is often in the spotlight of many diabetes research (Melnik, 2012).

**FIGURE 1:**
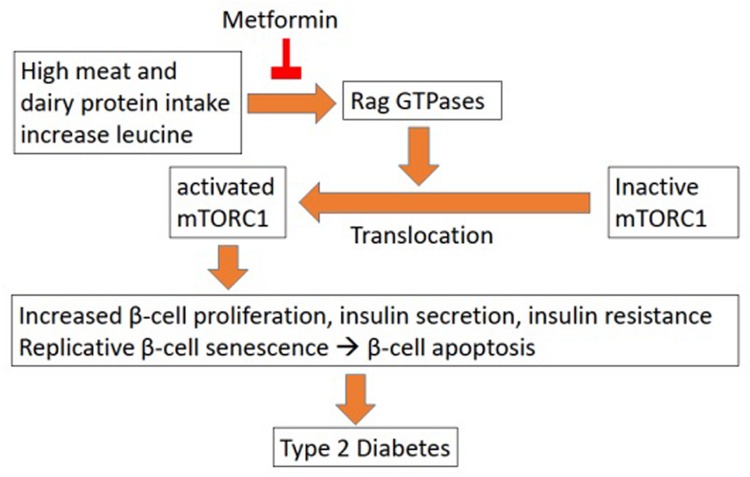
**Leucine influence on T2D (Melnik, 2012)**.

The relationship between leucine and glycaemic control is rather complex. In one way, it appears to help with the condition by stimulation of insulin production (Zhang et al., 2007). However, prolonged usage of excess leucine may accelerate the deterioration of the health condition of the patient (Melnik, 2012). Melnik (2012) surmised that excessive intake of leucine from meat (often red meat) leads to hyper activation of mTORC1 (mammalian target of rapamycin complex 1), a nutrient-sensitive kinase. mTORC1 when activated, leads to insulin resistance in the long term. While in the short term it will increase insulin production, prolonged supplement of leucine will cause pancreatic β-cells hyperfunction leading to their early senescence and apoptosis (**Figure 1**). The subsequent lack of insulin production will cause the increase of blood glucose as well as other side effects. In parallel, the primarily increased insulin production will also increase insulin resistance, which further complicates the condition.

However, Melnik (2012) were investigating how excess intake of leucine may contribute to T2D, whereas moderate intake of leucine may be beneficial toward T2D patients. Zhang et al. (2007) suggested that dietary supplement of leucine improves glucose and cholesterol metabolism, particularly decreasing hyperglycaemia and hypercholesterolemia in mice. Although the paper in question was mainly interested in obesity, there may be some indication that dietary leucine would help with diabetes. With high fat diet mice, having leucine in their water supply lowered the glucose content in their plasma sample after fasting. Although concentration of insulin was lower in high fat plus leucine fed mice, the paper claimed that these mice were more glucose tolerant and insulin sensitive. However, since these tests were done on healthy mice, more works are needed to test on diabetic mice.

Another study found that a mixture of protein hydrolysate and leucine increased insulin level and helped reduce glucose level in blood plasma (Manders et al., 2006). Their research indicated that while supplementing with additional leucine had a distinct advantage on insulin response from healthy subjects, distinction between the effects of protein supplement with or without leucine was lost in T2D subjects. It may be explained by T2D patients already having a decreased insulin sensitivity. In addition, postprandial glucose response indicated that both protein supplement with or without leucine supplement could effectively reduce glucose response in both healthy and T2D subjects. Therefore, the authors concluded that protein hydrolysate augments endogenous insulin secretion with or without additional leucine. However, the authors admitted that there are still more work to be done to be able to conclusively indicate that the role of leucine and its role with diabetes. They pointed out that healthy and diabetic subjects’ plasma glucose responses occur on different scales (i.e., diabetic subjects have higher plasma glucose responses). Also, they showed that glucose responses were inversely correlated with the accompanying insulin responses in patients with T2D. However, this was expected, for the insulin response in the T2D patient did not behave like that of control subjects, suggesting that glucose content measurement should be undertaken on diabetic patients in parallel to healthy subjects. However, the author maintained that incorporation of leucine should still be considered as beneficial toward improving the condition of T2D.

Although leucine’s role in blood glucose is somewhat ambiguous, its role in the positive regulation of mTORC1 is often mentioned (Manders et al., 2006; Melnik, 2012; Norton et al., 2012). As mentioned early on in this section, the induction of mTOCR1 induces insulin production and hence plasma glucose reduction. It seems to positively correlate with muscle growth, thus benefiting diabetic patients in terms of retaining muscle mass (Manders et al., 2006). Although Norton et al. (2012) suggest that the addition of extra protein from whey or added leucine increases plasma insulin, one has to keep in mind that this particular experiment has been conducted on healthy male mice with a short time scale. So the effect of persistent leucine supplement on diabetic subject is inconclusive. Yet it would be prudent to consider Melnik (2012) warning of long term detrimental effect of excessive leucine intake on pancreatic β-cells.

One may also need to consider the potential effect of leucine on different age groups. One study on advanced age male found that prolonged leucine supplement has no effect on glycaemic control or muscle mass augmentation (Leenders et al., 2011). Meanwhile in another study, a high proportion of leucine was required to stimulate the rate of muscle protein synthesis, particularly in elderly patients as compared to younger patients (Katsanos et al., 2006).

Over all, much work is required to be done on the subject of protein supplementation for patients with T2D. While protein and amino acids, particularly leucine, have many advantages in improving the condition of T2D, one has to consider its implication on pancreatic β-cell senescence subsequently increasing the dependency of externally supplied insulin. Therefore, when advising on dietary treatments, considerations of the present condition of the subject is important to keep in mind.

### Fat/Cholesterol

Fat is an essential part of a healthy diet. Many biological functions are dependent on fat but as with any other nutrients, problem arise when it is consumed in excess. In particular, it increases the risk of blocking blood vessels when these lipids enter into the blood stream, and the problem gets further aggravated when coupled with elevated level of glucose (American Heart Association, 2016). If not controlled carefully, over accumulation of fat and cholesterol will lead to an increasing risk of cardiovascular disease (CVD), one of the complications very commonly observed in diabetics. There are three well known cholesterol and fat, low-density lipoprotein cholesterol (LDL-C), high-density lipoprotein cholesterol (HDL-C), and triglycerides (TG).

Low-density lipoprotein cholesterol has been associated with higher risk for CVD (Marz et al., 2011). It can be caused by a diet high in *trans* fats often associated with industrially hydrogenated vegetable fat as well as from fat of grazing animals (de Souza et al., 2015; Ganguly and Pierce, 2015). The primary concern with LDL-C in terms of T2D is that it induces apoptosis in pancreatic β-cells *in vitro* (Abderrahmani et al., 2007). Also, Drew et al. (2009) has discovered in their experiment that higher oxidated LDL-C reduces insulin secretion in cultured mouse pancreatic β-cells. Studies show that it will require more than a year of sustained lifestyle intervention to improve the LDL-C profile (The Look Ahead Research Group, 2010; Wing et al., 2011). Therefore, lowering LDL-C is often considered as a target in management strategy.

High-density lipoprotein cholesterol has been associated with lowering the risk for CVD. Lowered HDL-C will put one at a higher risk of CVD, particularly people with T2D (The Look Ahead Research Group, 2010; Wing et al., 2011). It is particularly responsive to lifestyle intervention. There are evidences of HDL-C being able to reduce plasma glucose by intravenous reconstituted HDL-C (rHDL) to T2D patients (Drew et al., 2009). The results demonstrated that infusing rHDL into the patient’s blood stream led to an increase of β-cell function and subsequently plasma insulin level. In addition, a recent article pointed out that HDL-C may have a positive role in improving β-cell function as well as offering protection against stress-induced apoptosis (Kruit et al., 2010). While HDL-C level is generally a good health indicator, there is a scenario where HDL-C benefits may be inhibited. If the HDL-C is enriched with triglycerides and depletion in cholesteryl ester with conformational alternation of apolipoprotein A-1, this will render HDL-C more likely to be immobilized on arterial wall (Kontush and Chapman, 2008). Therefore, management strategy have to consider beyond HDL-C profile alone.

Triglycerides (TG) is known as the most common type of fat in the body. It is also known that high level of TG is not necessarily the cause of diabetes but an indicator of an individual at risk of diabetes (Dansigner, 2015). For an example, reducing TG has been found to reduce the risk or delay in the onset of T2D (Kruit et al., 2010). High level of TG have also been linked with higher incidence of cardiovascular complication, in particular coronary heart disease in an ethnically Japanese study (Sone et al., 2011). Therefore, health risk is often associated with high TG level.

Lipid improvement may not have a direct positive effect on glycaemic index, however, it has the potential to help in reducing the subsequent complications. As part of a review, Bitzur et al. (2009) concluded that omega-3 has been shown with the ability to regulate the balance of LDL, HDL, and TG. Therefore, it has been successful in correcting dyslipidemia, a condition that reflects an imbalance between LDL, HDL, and TG, and which is part of the T2D symptoms that can cause cardiovascular disease events. These are but a few examples on how diet may improve the condition of those that have T2D.

### Micro- and Anti-Nutrients

Micro-nutrients are nutrients such as minerals and vitamins that are required in small quantity while being essential for health (Karunasinghe et al., 2016; Maiti et al., 2016). Anti-nutrients are often referred to as those that decrease the digestibility of nutrients (Pal et al., 2016). Little quantification has been made when it comes to dietary recommendation on micro-nutrients and anti-nutrients and their effects on T2D.

Several metabolic pathways and cellular reactions in the body require minerals and vitamins to act as coenzymes and cofactors. Unlike the previously established notion that their deficiencies are related to specific diseases, with the progress in nutritional biology research, it has increasingly become clear that these micro-nutrients also have the potential to impact on other chronic ailments such as Types 1 and Type 2 diabetes (Mooradian et al., 1994; Franz and Bantle, 1999). Indeed, reports confirm that micro-nutrients play a part in improving the diabetic condition (Thorne et al., 1983; Boivin et al., 1988; O’Connell, 2001; McDougall et al., 2005; He et al., 2007).

Meanwhile, anti-nutrients research has shown that α-amylase can be inhibited by various plant-derived molecules like luteolin, polyphenols, as well as amylase inhibitors (Boivin et al., 1988; McDougall et al., 2005; He et al., 2007). In particular, Boivin et al. (1988) showed that α-amylase inhibitor can significantly reduce postprandial glucose peak in both healthy and T2D subjects. To deter predation, plant α-amylase inhibitor causes the reduction of starch digestion by acting against animal α-amylase activity (Singh et al., 2010). However, these inhibitors can be inactivated/reduced by heat during cooking (Rehman and Shah, 2005). Polyphenols from tea can also help with reducing starch, lipid, and protein bioavailability, thus reducing excessive amount of nutrients to be absorbed by the body (He et al., 2007). Others like anthocyanin which is often found in soft fruits inhibit α-glucosidase activities (McDougall et al., 2005). This can be an effective tool to combat T2D, as diabetes is often caused by overindulgence in food.

In terms of minerals, the best established beneficial effector for diabetics is supplemental chromium (Cr) (Anderson, 1998; Anderson, 2000; Sharma et al., 2011; Yin and Phung, 2015). Diabetics may not show any specific Cr deficiency (Anderson, 1998), but Cr basically acts by creating a better mechanism for insulin action through improved receptor numbers, binding ability, phosphorylation and activation (O’Connell, 2001). The indirect nature of Cr’s benefit toward T2D may be the cause of some contradictory results among studies (Gunton et al., 2005). In its naturally occurring form, Cr is the active component of glucose tolerance factor (GTF), which renders Cr into the most bioavailable and it is safer than its other supplemental forms like chromium picolinate (Vincent, 2007). Consuming 100 microgram of GTF Cr by consuming whole grains, beans, nuts/seeds, and mushrooms has been proposed to significantly alleviate diabetes. Apart from Cr, minerals like zinc, magnesium, manganese, potassium, and vanadium have also been found to be essential for T2D patients by controlling glucose and insulin homeostasis (O’Connell, 2001; Diabetes.co.uk, 2016d). Mineral deficiency interferes with the functioning of insulin, thus affecting the glucose metabolism, and deregulating blood sugar content.

Besides minerals, vitamins also regulate the activity of insulin and thus have been promoted as role players in diabetes management (Pittas et al., 2006, 2007; Pflipsen et al., 2009). In its most bioavailable and natural form, vitamin E (d-α-tocopherol) was found to significantly improve glycaemic control without changing insulin secretion (Paolisso et al., 1993). Vitamin B12 deficiency has been found to be prevalent (22%) in T2D populations (Pflipsen et al., 2009). Furthermore, patients who have diabetes-related CVD also develop the mortality risk factor, hyperhomocysteinemia (Hhcys), characterized by very high total homocysteine present in the blood plasma leading to death. Vitamin B6, folic acid (vitamin B9), and vitamin B12 have been used to decrease levels of plasma homocysteine and the risk of CVD in Type 2 diabetics (Thornalley and Rabbani, 2010). Molecules such as inositol, coenzyme Q10 and carnitine are regulators of various carbohydrate, fatty acid, and protein metabolism pathways. Although their direct role in diabetes is still unclear, they may be useful to prevent or help in several diabetes-associated effects like diabetic ketoacidosis, diabetic retinopathy, and diabetic neuropathy (Diabetes.co.uk, 2016d). Thus, regular consumption of micro-nutrients in the form of natural or fortified food or any other intervention strategies supplementing micro-nutrients should be part of diabetes management.

### Research on Food Processing/Cooking Methods

Another aspect to consider in dietary intervention is that most foods consumed are processed or cooked. For example, the cooking methods as well as the duration and level of heat used in cooking may change the ratio of different starch fractions (Snow and O’Dea, 1981; Anderson and Guraya, 2006; Roopa and Premavalli, 2008; Chung and Liu, 2009; Zhang et al., 2009). In addition, the subsequent storage/refrigeration of food has also been shown to have effects on reduced RDS content (Mishra et al., 2008). There are some inconsistencies though in such findings. For an example, a report stated that heating with microwaves have no significant effect on starch digestibility (Anderson and Guraya, 2006). While Zhang et al. (2009) showed a different conclusion where heating has been related with increased RS. Therefore subsequently, cooking and processing will have an impact to GI value (Frei et al., 2003; Hu et al., 2004). It is also important to know that dietary fiber content, tannin and *in vitro* protein digestibility of processed grain, all affected by cooking and processing, can affect GI (Pushparaj and Urooj, 2011).

For an example, grain such as pearl millet is never eaten raw or as a whole grain. It is milled with the seed coat (rich in dietary fiber and micro-nutrients) to prepare whole meal flour utilized in preparation of foods. Commonly used traditional methods of processing and cooking pearl millet are: milling, roasting, boiling, pressure cooking, sprouting/germination. Processes such as fermentation and germination also are known to decrease the phytate (an anti-nutrient) content by 60% and improve bio-availability of minerals (Suma and Urooj, 2014). Processing such as dehulling has been found to cause a significant reduction in protein, polyphenol and phytic acid content (Pawar and Parlikar, 1990). Fermentation after dehulling can also cause a significant increase in the *in vitro* protein digestibility (IVPD) from 3 to 14% (El Hag et al., 2002). However, cooking had little effects on the total dietary fiber (TDF; Pushparaj and Urooj, 2011). When put together, all these processes will cause changes in the availability of various nutrients content and should be considered as part of the management program.

There is much advice available on diabetics diet, but little advice in terms of scientific research on methods of processing/cooking that are most beneficial for diabetics. Some suggestions such as a research on pressure cooked legume have found the increase of SDS content at the expense of RDS and RS (Kasote et al., 2014). While the authors have suggested that it would benefit people who are suffering from diabetes, one problem as they have pointed out, that the predicted GI (pGI) is higher after pressure cooking, which make it less desirable for diabetics. However, while the SDS, RS and RDS analysis was well defined in the paper, their pGI was not. Other studies from rice and millet have also shown increased SDS content after heat moisture treatment supporting the potential benefit of cooked grain for diabetics (Anderson and Guraya, 2006; Lehmann and Robin, 2007).

## Traits Desired in Plants for Diabetic Dietary Requirement

### Plant Modification (Breeding and Transgenic) Toward Targeted Phenotype

Traditional plant breeding has played a key role in improving plant yield and vigor and recently its emphasis is shifting to improving nutritional quality too (Khoshgoftarmanesh et al., 2010; Richards et al., 2010). One of the prime example is the breeding of soybean (Clarke and Wiseman, 2000). As Clarke and Wiseman (2000) report, there have been a number of efforts in breeding soybean to reduce its anti-nutrients content. By reducing various anti-nutrients compounds the protein availability in food for human or feed for farm animals has been substantially improved in this crop. This sets an excellent example of improving nutritional quality of the crop through selective breeding.

Efficiency of selective breeding can be further improved using molecular markers (Yadav et al., 2011, 2013; Varshney et al., 2013; Thudi et al., 2014; Kale et al., 2015). Marker-assisted breeding has been developed recently by identifying genomic regions or even causational genes that led to the development of better crop in terms of resilience, crop value, and nutritional qualities (Serraj et al., 2005; Yadav et al., 2011; Varshney et al., 2013, 2014; Thudi et al., 2014). With new marker-assisted technologies, breeding becomes less time consuming than traditional breeding and will continue to be a major tool for crop development and improvement.

There are many markers developed to identify crops with abiotic/biotic stress resistance and other agronomic traits including yield (Fan et al., 2006; Jena and Mackill, 2008; Miedaner and Korzun, 2012; Sharma et al., 2014). There are also efforts that identified markers showing implication in nutrition (Muthamilarasana et al., 2016). These markers targets include proteins (Kumar et al., 2011), minerals (Zhang et al., 2011; Kwon et al., 2012), sugar/acid (Francia et al., 2005) as well as anti-oxidants and phenolics (Shao et al., 2010; Torres et al., 2010) in various crops. Markers that have implication on downstream food processing such as malting for barley and cooking for rice are also available (Francia et al., 2005). Collection of such markers will allow faster developmental time for producing desirable nutritional traits in various crops.

Various diabetic associations have identified micro-nutrients and anti-nutrients that influence the speed of digestion or optimizing nutrition use. Fortifying plants with micro-nutrients have been the focus of much research to improve nutritional quality to combat diabetes as well as malnutrition (O’Connell, 2001; Bouis and Welch, 2010). Much has been done to improve micro-nutrients content (Cakmak et al., 2010) but more can be done in targeting marco-nutritional benefits (e.g., carbohydrate, fat, and protein) for diabetics.

Although less welcomed in the public domain, transgenic technology is another mean and tool for biofortification of grain nutritional traits (Pérez-Massot et al., 2012; Galili and Amir, 2013). Galili and Amir (2013) argued that traditional breeding methods have failed to raise some aspects of nutrition in crop to a satisfactory level. With continually expending knowledge and understanding of the biochemical pathways with molecular control, as well as with improvement of the transgenic technique, one can argue that genetic modification should be considered as a viable option. On the other hand, even though transgenic technology may have its advantages over traditional breeding, it has its limitations. For an example, one has to consider the acceptance of the general public, as well as other considerations beyond the technology itself (Sample, 2012). Examples would be the legal frame work for this technology to be used on market products as well as the regulatory framework to conduct these processes safely. Currently, the additional cost to secure transgenic trial sites in Europe can exceed the cost of the experiment itself due to the lack of public support (Cressey, 2012; Kuntz, 2012; The Telegraph, 2015). This in itself has much to desire as there is little evidence to assess the safety of such technology due to the lack of public support to explore and test such technology (Nature Editorial, 2012). Many experiments, including those for safety assessment, have suffered premature termination (Pilate et al., 2002; Kuntz, 2012). Perhaps much transparent process, public education, and campaign are needed to win over the trust of the general public if this technology is to be successfully deployed.

Over all, based on the above discussion on nutrition requirements of diabetics, some of the targeted benefits of improving staple crops for nutritional traits are clear. Given that there already exist technologies for crop improvement, we can start looking more into improving nutritional qualities such as lipid, fiber, mineral, amino acid and starch contents in our staple crops.

### Millets Benefit for Diabetics

There are many dietary advice and options readily available for diabetics. Some have even provided advice on food groups down to grain type (Dansinger, 2016). Recently, millets are receiving increasing spotlight in combating diabetes as a dietary option (Henry and Kaur, 2014; Nambiar and Patwardhan, 2014; Muthamilarasana et al., 2016). Indeed, there are evidences to support that millets have many properties making it a good dietary option for diabetics. For an example, an experiment that has used diabetic mice to test different diets has concluded that added millet protein can increase insulin sensitivities, and reduce blood glucose level as well as triglyceride level (Nishizawa et al., 2009). Added benefits such as increased plasma level of adiponectin and high-density lipoprotein cholesterol were also found in their 3 weeks study.

The cereal crop millet is one of the most abundant crops grown in India and African continent, and provides a staple food for many poor communities (Ravi, 2004). Compared to other cereal crops such as wheat and maize, millets are high in nutritional content, gluten free, and have low GI (Abdalla et al., 1998). They provide high energy, high dietary fiber, protein with balanced amino acid profile, many essential minerals, some vitamins, and antioxidants (FAO, 1995; Lestienne et al., 2005; Suma and Urooj, 2012). These play a substantial role in prevention of many human illnesses such as T2D, cancer, cardiovascular, and neurodegenerative diseases (Kannan, 2010; Shahidi and Chandrasekara, 2013). There is great potential for harnessing these positive attributes through selective breeding. Subsequently, combining grain processing/cooking methods and food production technologies in producing food product from such varieties will be directly useful for controlling diabetes through diets.

There are various species of millets (pearl, foxtail, finger, little and kodo, just to name a few) growing in various parts of the world (Ravi, 2004). These millets are known to be able to survive and produce food in regions that are more prone to drought. The added benefit for millets is their potential positive contribution toward controlling the symptoms of diabetes (Choi et al., 2005; Park et al., 2008; Shobana et al., 2010; Jali et al., 2012). They are known to have higher SDS (Liu et al., 2006), mineral (FAO, 1995) as well as leucine (Ejeta et al., 1987; FAO, 1995) contents, that are positively attributed toward healthy diet for diabetics. Furthermore well characterized genetic, genomic and breeding methods exist for pearl millet (Yadav et al., 2011; Hash et al., 2003), providing a head start for breeding program in this crop.

Amongst millets antidiabetic properties, a study in India reported that patients with T2D fed with foxtail millet for 90 days showed improved glycaemic control as well as other improvements (Jali et al., 2012). The patients were given the diet of a combination of foxtail millet, split black gram and spice mix with a high degree of compliance. The result showed a reduced HbA1c, fasting glucose, insulin, total cholesterol, triglyceride, and LDL concentrations. These were all indications that this diet had a positive impact on T2D patients. The reduction of cholesterol, triglyceride and LDL-C concentration had a positive implication in cardio health (Marz et al., 2011; Sone et al., 2011; Wing et al., 2011), a complication that many T2D patients suffered from (Bitzur et al., 2009). One could argue that the effect of medication or the more regulated diet could cause an equal positive impact. However, millet’s positive role was consistent throughout these studies. Further, medication has negative side-effects that diet did not introduce.

Similar research on effects of finger millet on T2D rat had also been published (Shobana et al., 2010). The study demonstrated that finger millet may help reduce subcapsular cataract when T2D mice were fed with added finger millet seeds coat. In this experiment, Shobana et al. (2010) had also observed the reversal of hypercholesterolemia and hypertriacylglycerolemia associated with diabetes. Not only did finger millet had an implication in diabetics’ health but health improvement in general, as many obese rat subjects had experienced weight loss.

Two studies from the same group on proso- millet and foxtail millet concluded that diet with mixture of their respective protein faction improved HDL-C concentration as well as reduced insulin and plasma glucose concentration (Choi et al., 2005; Park et al., 2008). Though these were positive results in managing diabetes, caution have to be exercised that such results were drawn from protein fraction only and not the whole grain. The more wholesome properties of the grain such as fat and starch portion had yet to be investigated. This did not, however, negate the fact that these millets carried beneficial properties toward managing diabetes.

Thus, while there are many indications of dietary health benefits offered by millets in managing diabetes, more research is required.

## Concluding Remarks/Summary

Much is desired toward developing crop products that can offer options to treat diabetes through diet. Even more research is needed for developing crop that can be used as a raw materials for these dietary products. In light of their benefits, millets hold a key to the well-being for those who suffer from, and those that are at risk of, diabetes. More research must be done in establishing the benefit and the method of deployment of these benefits in combating the global rising tide of diabetes.

## Author Contributions

All authors listed, have made substantial, direct and intellectual contribution to the work, and approved it for publication.

## Conflict of Interest Statement

The authors declare that the research was conducted in the absence of any commercial or financial relationships that could be construed as a potential conflict of interest. The reviewer EP and handling Editor declared their shared affiliation, and the handling Editor states that the process nevertheless met the standards of a fair and objective review.

## Footnotes

1 http://www.who.int/diabetes/en/

## References

[B1] Abdalla A. A. El Tinay A. H. Mohamed B. E. Abdalla A. H. (1998). Proximate composition, starch, phytate and mineral contents of 10 pearl millet genotypes. *Food Chem.* 63 243–246. 10.1016/S0308-8146(97)00194-5

[B2] Abderrahmani A. Niederhauser G. Favre D. Abdelli S. Ferdaoussi M. Yang J. Y. (2007). Human high-density lipoprotein particles prevent activation of the JNK pathway induced by human oxidised low-density lipoprotein particles in pancreatic beta cells. *Diabetologia* 50 1304–1314. 10.1007/s00125-007-0642-z 17437081

[B3] Agama-Acevedo E. Islas-Hernández J. J. Pacheco-Vargas G. Osorio-Díaz P. Bello-Pérez L. A. (2012). Starch digestibility and glycemic index of cookies partially substituted with unripe banana flour. *LWT - Food Sci. Technol.* 46 177–182. 10.1016/j.lwt.2011.10.010

[B4] Ajala O. English P. Pinkney J. (2013). Systematic review and meta-analysis of different dietary approaches to the management of type 2 diabetes. *Am. J. Clin. Nutr.* 97 505–516. 10.3945/ajcn.112.042457 23364002

[B5] American Diabetes Association (2015a). *Diabetes Myths.* Available at: http://www.diabetes.org/diabetes-basics/myths/

[B6] American Diabetes Association (2015b). *Hypoglycemia (Low Blood Glucose).* Available at: http://www.diabetes.org/living-with-diabetes/treatment-and-care/blood-glucose-control/hypoglycemia-low-blood.html

[B7] American Diabetes Association (2016). *The Mediterranean Diet - What’s the Story.* Available at: http://www.diabetes.org/mfa-recipes/tips/2011-09/featured-article-the.html

[B8] American Heart Association (2016). *Cholesterol Abnormalities and Diabetes.* Available at: http://www.heart.org/HEARTORG/Conditions/Diabetes/WhyDiabetesMatters/Cholesterol-Abnormalities-Diabetes_UCM_313868_Article.jsp#.Vqsyl1Lz5SA.

[B9] Anderson A. K. Guraya H. S. (2006). Effects of microwave heat-moisture treatment on properties of waxy and non-waxy rice starches. *Food Chem.* 97 318–323. 10.1016/j.foodchem.2005.04.025

[B10] Anderson R. A. (1998). Chromium, glucose intolerance and diabetes. *J. Am. Coll. Nutr.* 17 548–555. 10.1080/07315724.1998.10718802 9853533

[B11] Anderson R. A. (2000). Chromium in the prevention and control of diabetes. *Diab. Metab. (Paris)* 26 22–27. 10705100

[B12] Avena N. M. Rada P. Hoebel B. G. (2008). Evidence for sugar addiction: behavioral and neurochemical effects of intermittent, excessive sugar intake. *Neurosci. Biobehav. Rev.* 32 20–39. 10.1016/j.neubiorev.2007.04.019 17617461 PMC2235907

[B13] Bassil M. S. Gougeon R. (2013). Muscle protein anabolism in type 2 diabetes. *Curr. Opin. Clin. Nutr. Metab. Care* 16 83–88. 10.1097/MCO.0b013e32835a88ee 23196814

[B14] Ben-Avraham S. Harman-Boehm I. Schwarzfuchs D. Shai I. (2009). Dietary strategies for patients with type 2 diabetes in the era of multi-approaches; review and results from the Dietary Intervention Randomized Controlled Trial (DIRECT). *Diabetes.* *Res. Clin. Pract.* 86(Suppl. 1) S41–S48. 10.1016/j.diabres.2009.08.011 20115931

[B15] Bitzur R. Cohen H. Kamari Y. Shaish A. Harats D. (2009). Triglycerides and HDL Cholesterol: stars or second leads in diabetes? *Diabetes Care* 32 S373–S377. 10.2337/dc09-er02 19875584 PMC2811435

[B16] Boivin M. Flourie B. Rizza R. A. Go V. L. DiMagno E. P. (1988). Gastrointestinal and metabolic effects of amylase inhibition in diabetics. *Gastroenterology* 94 387–394. 10.1016/0016-5085(88)90426-X 2446948

[B17] Bouis H. E. Welch R. M. (2010). Biofortification—a sustainable agricultural strategy for reducing micronutrient malnutrition in the global south. *Crop Sci.* 50 S20–S32. 10.2135/cropsci2009.09.0531

[B18] Boulton A. J. M. Vinik A. I. Arezzo J. C. Bril V. Feldman E. L. Freeman R. (2005). Diabetic neuropathies: a statement by the american diabetes association. *Diabetes Care* 28 956–962. 10.2337/diacare.28.4.956 15793206

[B19] Brinkworth G. D. Noakes M. Parker B. Foster P. Clifton P. M. (2004). Long-term effects of advice to consume a high-protein, low-fat diet, rather than a conventional weight-loss diet, in obese adults with type 2 diabetes: one-year follow-up of a randomised trial. *Diabetologia* 47 1677–1686. 10.1007/s00125-004-1511-7 15480538

[B20] Brownlee M. (2001). Biochemistry and molecular cell biology of diabetic complications. *Nature* 414 813–820. 10.1038/414813a 11742414

[B21] Cakmak I. Pfeiffer W. H. McClafferty B. (2010). REVIEW: biofortification of durum wheat with zinc and iron. *Cereal Chem. J.* 87 10–20. 10.1094/CCHEM-87-1-0010

[B22] Chandalia M. Garg A. Lutjohann D. von Bergmann K. Grundy S. M. Brinkley L. J. (2000). Beneficial effects of high dietary fiber intake in patients with Type 2 diabetes mellitus. *New Engl. J. Med.* 342 1392–1398. 10.1056/NEJM200005113421903 10805824

[B23] Choi Y.-Y. Osada K. Ito Y. Nagasawa T. Choi M.-R. Nishizawa N. (2005). Effects of dietary protein of korean foxtail millet on plasma adiponectin, HDL-cholesterol, and insulin levels in genetically Type 2 diabetic mice. *Biosci. Biotechnol. Biochem.* 69 31–37. 10.1271/bbb.69.31 15665464

[B24] Chung H. J. Liu Q. (2009). Effect of gamma irradiation on molecular structure and physicochemical properties of corn starch. *J. Food Sci.* 74 C353–C361. 10.1111/j.1750-3841.2009.01224.x 19646027

[B25] Clarke E. J. Wiseman J. (2000). Developments in plant breeding for improved nutritional quality of soya beans I. Protein and amino acid content. *J. Agric. Sci.* 134 111–124. 10.1017/S0021859699007431

[B26] Coppell K. J. Kataoka M. Williams S. M. Chisholm A. W. Vorgers S. M. Mann J. I. (2010). Nutritional intervention in patients with type 2 diabetes who are hyperglycaemic despite optimised drug treatment—Lifestyle Over and Above Drugs in Diabetes (LOADD) study: randomised controlled trial. *BMJ* 341:c3337 10.1136/bmj.c3337 PMC2907481 20647285

[B27] Cressey D. (2012). *Rothamstedtrail Attacked.* Available at: http://blogs.nature.com/news/2012/05/rothamsted-gm-trial-attacked.html

[B28] Crozier S. J. Kimball S. R. Emmert S. W. Anthony J. C. Jefferson L. S. (2005). Oral leucine administration stimulates protein synthesis in rat skeletal muscle. *J. Nutr.* 135 376–382. 15735066 10.1093/jn/135.3.376

[B29] Dansigner M. (2015). *How Triglycerides Affect Your Risk of Diabetes.* Available at: http://www.webmd.com/cholesterol-management/diabetes

[B30] Dansinger M. (2016). *Best and Worst Food for Diabetes.* Available at: http://www.webmd.com/diabetes/diabetic-food-list-best-worst-foods

[B31] Dardevet D. Sornet C. Balage M. Grizard J. (2000). Stimulation of in vitro rat muscle protein synthesis by leucine decreases with age. *J. Nutr.* 130 2630–2635. 11053498 10.1093/jn/130.11.2630

[B32] de Souza R. J. Mente A. Maroleanu A. Cozma A. I. Ha V. Kishibe T. (2015). Intake of saturated and trans unsaturated fatty acids and risk of all cause mortality, cardiovascular disease, and type 2 diabetes: systematic review and meta-analysis of observational studies. *BMJ* 351:h3978 10.1136/bmj.h3978 PMC4532752 26268692

[B33] Diabetes.co.uk (2016a). *Juice and Diabetes.* Available at: http://www.diabetes.co.uk/food/juice-and-diabetes.html

[B34] Diabetes.co.uk (2016b). *Mediterranean Diet.* Available at: http://www.diabetes.co.uk/diet/mediterranean-diet.html

[B35] Diabetes.co.uk (2016c). *Type 1 Diabetes.* Available at: http://www.diabetes.co.uk/type1-diabetes.html

[B36] Diabetes.co.uk (2016d). *Vitamins and Minerals.* Available at: http://www.diabetes.co.uk/vitamins-supplements.html

[B37] Diabetes Prevention Program Research Group (2002). Reduction in the incidence of Type 2 diabetes with lifestyle intervention or metformin. *New Engl. J. Med.* 346 393–403. 10.1056/NEJMoa012512 11832527 PMC1370926

[B38] Diabetes U. K. (2016). *Myth: Sugar Causes Diabetes.* Available at: https://www.diabetes.org.uk/Guide-to-diabetes/Enjoy-food/Eating-with-diabetes/Diabetes-food-myths/Myth-sugar-causes-diabetes/

[B39] Drew B. G. Duffy S. J. Formosa M. F. Natoli A. K. Henstridge D. C. Penfold S. A. (2009). High-density lipoprotein modulates glucose metabolism in patients with Type 2 diabetes mellitus. *Circulation* 119 2103–2111. 10.1161/CIRCULATIONAHA.108.843219 19349317

[B40] Ejeta G. Hassen M. M. Mertz E. T. (1987). In vitro digestibility and amino acid composition of pearl millet (*Pennisetum typhoides*) and other cereals. *Proc. Natil. Acad. Sci. U.S.A.* 84 6016–6019. 10.1073/pnas.84.17.6016 PMC298998 3476923

[B41] El Hag M. E. El Tinay A. H. Yousif N. E. (2002). Effect of fermentation and dehulling on starch, total polyphenols, phytic acid content and in vitro protein digestibility of pearl millet. *Food Chem.* 77 193–196. 10.1016/S0308-8146(01)00336-3

[B42] Elliott S. S. Keim N. L. Stern J. S. Teff K. Havel P. J. (2002). Fructose, weight gain, and the insulin resistance syndrome. *Am.* *J. Clin. Nutr.* 76 911–922. 10.1093/ajcn/76.5.911 12399260

[B43] Englyst H. N. Kingman S. M. Cummings J. H. (1992). Classification and measurement of nutritionally important starch fractions. *Eur.* *J. Clin. Nutr.* 46(Suppl. 2) S33–S50. 1330528

[B44] Ezeogu L. I. Duodu K. G. Emmambux M. N. Taylor J. R. N. (2008). Influence of cooking conditions on the protein matrix of sorghum and maize endosperm flours. *Cereal Chem. J.* 85 397–402. 10.1094/CCHEM-85-3-0397

[B45] Fan Z. Robbins M. D. Staub J. E. (2006). Population development by phenotypic selection with subsequent marker-assisted selection for line extraction in cucumber (*Cucumis sativus* L.). *Theor. Appl. Genet.* 112 843–855. 10.1007/s00122-005-0186-x 16397790

[B46] FAO (1995). *Sorghum and Miillets in Human Nutrition.* Available at: www.fao.org/docrep/t0818e/T0818E0e.htm#Minerals

[B47] Francia E. Tacconi G. Crosatti C. Barabaschi D. Bulgarelli D. Dall’Aglio E. (2005). Marker assisted selection in crop plants. *Plant Cell Tissue Organ Cult.* 82 317–342. 10.1007/s11240-005-2387-z

[B48] Franz M. J. Bantle J. P. (1999). *American Diabetes Association Guide to Medical Nutrition Therapy for Diabetes.* Alexandria: American Diabetes Association.

[B49] Franz M. J. Powers M. A. Leontos C. Holzmeister L. A. Kulkarni K. Monk A. (2010). The evidence for medical nutrition therapy for Type 1 and Type 2 diabetes in adults. *J.* *Am. Diet. Assoc.* 110 1852–1889. 10.1016/j.jada.2010.09.014 21111095

[B50] Frei M. Siddhuraju P. Becker K. (2003). Studies on the in vitro starch digestibility and the glycemic index of six different indigenous rice cultivars from the Philippines. *Food Chem.* 83 395–402. 10.1016/S0308-8146(03)00101-8

[B51] Gaede P. Vedel P. Parving H. H. Pedersen O. (1999). Intensified multifactorial intervention in patients with type 2 diabetes mellitus and microalbuminuria: the Steno type 2 randomised study. *Lancet* 353 617–622. 10.1016/S0140-6736(98)07368-1 10030326

[B52] Galili G. Amir R. (2013). Fortifying plants with the essential amino acids lysine and methionine to improve nutritional quality. *Plant Biotechnol. J.* 11 211–222. 10.1111/pbi.12025 23279001

[B53] Gallant D. J. Bouchet B. Buleon A. Perez S. (1992). Physical characteristics of starch granules and susceptibility to enzymatic degradation. *Eur.* *J. Clin. Nutr.* 46(Suppl. 2) S3–S16. 1330527

[B54] Ganguly R. Pierce G. N. (2015). The toxicity of dietary trans fats. *Food Chem. Toxicol.* 78 170–176. 10.1016/j.fct.2015.02.004 25684416

[B55] Greenwood D. C. Threapleton D. E. Evans C. E. L. Cleghorn C. L. Nykjaer C. Woodhead C. (2013). Glycemic index, glycemic load, carbohydrates, and Type 2 diabetes: systematic review and dose–response meta-analysis of prospective studies. *Diabetes Care* 36 4166–4171. 10.2337/dc13-0325 24265366 PMC3836142

[B56] Grysman A. Carlson T. Wolever T. M. (2008). Effects of sucromalt on postprandial responses in human subjects. *Eur.* *J. Clin. Nutr.* 62 1364–1371. 10.1038/sj.ejcn.1602890 17717534

[B57] Gunton J. E. Cheung N. W. Hitchman R. Hams G. O’Sullivan C. Foster-Powell K. (2005). Chromium supplementation does not improve glucose tolerance, insulin sensitivity, or lipid profile: a randomized, placebo-controlled, double-blind trial of supplementation in subjects with impaired glucose tolerance. *Diabetes Care* 28 712–713. 10.2337/diacare.28.3.712 15735214

[B58] Halvatsiotis P. Short K. R. Bigelow M. Nair K. S. (2002). Synthesis rate of muscle proteins, muscle functions, and amino acid kinetics in type 2 diabetes. *Diabetes Metab.* *Res. Rev.* 51 2395–2404. 10.2337/diabetes.51.8.2395 12145150

[B59] Hash C. T. Bhasker Raj A. G. Lindup S. Sharma A. Beniwal C. R. Folkertsma R. T. (2003). Opportunities for marker-assisted selection (MAS) to improve the feed quality of crop residues in pearl millet and sorghum. *Field Crops Res.* 84 79–88. 10.1016/S0378-4290(03)00142-4

[B60] Havel P. J. (2005). Dietary fructose: implications for dysregulation of energy homeostasis and lipid/carbohydrate metabolism. *Nutr.* *Rev.* 63 133–157. 10.1301/nr.2005.may.133-157 15971409

[B61] He Q. Lv Y. Yao K. (2007). Effects of tea polyphenols on the activities of α-amylase, pepsin, trypsin and lipase. *Food Chem.* 101 1178–1182. 10.1016/j.foodchem.2006.02.024

[B62] Hein G. Weiss C. Lehmann G. Niwa T. Stein G. Franke S. (2006). Advanced glycation end product modification of bone proteins and bone remodelling: hypothesis and preliminary immunohistochemical findings. *Ann.* *Rheum. Dis.* 65 101–104. 10.1136/ard.2004.034348 PMC1797982 16344492

[B63] Hennessy M. (2013). *Leucine: Where Whey Protein Gets its Magic.* Available at: http://www.nutraingredients-usa.com/Markets/Leucine-Where-whey-protein-gets-its-magic

[B64] Henry C. J. Kaur B. (2014). Diet-based management and treatment of diabetes. *World Clin. Diabetol.* 1 1–19.

[B65] Hu P. Zhao H. Duan Z. Linlin Z. Wu D. (2004). Starch digestibility and the estimated glycemic score of different types of rice differing in amylose contents. *J. Cereal Sci.* 40 231–237. 10.1016/j.jcs.2004.06.001

[B66] Jakubowicz D. Froy O. (2013). Biochemical and metabolic mechanisms by which dietary whey protein may combat obesity and Type 2 diabetes. *J.* *Nutr. Biochem.* 24 1–5. 10.1016/j.jnutbio.2012.07.008 22995389

[B67] Jali M. V. Kamatar M. Y. Jali S. M. Hiremath M. B. Naik R. K. (2012). Efficacy of value added foxtail millet therapeutic food in the management of diabetes and dyslipidamea in type 2 diabetic patients. *Recent Res. Sci. Technol.* 4 3–4.

[B68] Jena K. K. Mackill D. J. (2008). Molecular markers and their use in marker-assisted selection in rice. *Crop Sci.* 48 147–168. 10.2135/cropsci2008.02.0082

[B69] Jenkins D. J. Thorne M. J. Wolever T. M. Jenkins A. L. Rao A. V. Thompson L. U. (1987). The effect of starch-protein interaction in wheat on the glycemic response and rate of in vitro digestion. *Am.* *J. Clin. Nutr.* 45 946–951. 10.1093/ajcn/45.5.946 3578096

[B70] Jenkins D. J. Wolever T. M. Leeds A. R. Gassull M. A. Haisman P. Dilawari J. (1978). Dietary fibres, fibre analogues, and glucose tolerance: importance of viscosity. *BMJ* 1 1392–1394. 10.1136/bmj.1.6124.1392 647304 PMC1604761

[B71] Johnston K. L. Thomas E. L. Bell J. D. Frost G. S. Robertson M. D. (2010). Resistant starch improves insulin sensitivity in metabolic syndrome. *Diabet. Med.* 27 391–397. 10.1111/j.1464-5491.2010.02923.x 20536509

[B72] Kahleova H. Matoulek M. Malinska H. Oliyarnik O. Kazdova L. Neskudla T. (2011). Vegetarian diet improves insulin resistance and oxidative stress markers more than conventional diet in subjects with Type 2 diabetes. *Diabet.* *Med.* 28 549–559. 10.1111/j.1464-5491.2010.03209.x PMC3427880 21480966

[B73] Kale S. M. Jaganathan D. Ruperao P. Chen C. Punna R. Kudapa H. (2015). Prioritization of candidate genes in “QTL-hotspot” region for drought tolerance in chickpea (*Cicer arietinum* L.). *Sci. Rep.* 5:15296 10.1038/srep15296 PMC4609953 26478518

[B74] Kannan S. (2010). Finger millet in nutrition transition: an infant weaning food ingredient with chronic disease preventive potential. *Br. J. Nutr.* 104 1733–1734. 10.1017/S0007114510002989 20673383

[B75] Karunasinghe N. Zhu S. Ferguson L. R. (2016). Benefits of selenium supplementation on leukocyte DNA integrity interact with dietary micronutrients: a short communication. *Nutrients* 8:249 10.3390/nu8050249 PMC4882662 27128937

[B76] Kasote D. Nilegaonkar S. Agte V. (2014). Effect of different processing methods on resistant starch content and in vitro starch digestibility of some common indian pulses. *J. Sci. Ind. Res.* 73 541–546.

[B77] Katsanos C. S. Kobayashi H. Sheffield-Moore M. Aarsland A. Wolfe R. R. (2006). A high proportion of leucine is required for optimal stimulation of the rate of muscle protein synthesis by essential amino acids in the elderly. *Am. J. Physiol. Endocrinol. Metab.* 291 E381–E387. 10.1152/ajpendo.00488.2005 16507602

[B78] Khoshgoftarmanesh A. H. Schulin R. Chaney R. L. Daneshbakhsh B. Afyuni M. (2010). Micronutrient-efficient genotypes for crop yield and nutritional quality in sustainable agriculture. A review. *Agron. Sustain. Dev.* 30 83–107. 10.1051/agro/2009017

[B79] Kontush A. Chapman M. J. (2008). Why is HDL functionally deficient in type 2 diabetes? *Curr. Diab. Rep.* 8 51–59. 10.1007/s11892-008-0010-5 18366999

[B80] Kruit J. K. Brunham L. R. Verchere C. B. Hayden M. R. (2010). HDL and LDL cholesterol significantly influence beta-cell function in type 2 diabetes mellitus. *Curr.* *Opin. Lipidol.* 21 178–185. 10.1097/MOL.0b013e328339387b 20463468

[B81] Kumar J. Jaiswal V. Kumar A. Kumar N. Mir R. R. Kumar S. (2011). Introgression of a major gene for high grain protein content in some Indian bread wheat cultivars. *Field Crops Res.* 123 226–233. 10.1016/j.fcr.2011.05.013

[B82] Kuntz M. (2012). Destruction of public and governmental experiments of GMO in Europe. *GM Crops Food* 3 258–264. 10.4161/gmcr.21231 22825391

[B83] Kwon S.-J. Brown A. F. Hu J. McGee R. Watt C. Kisha T. (2012). Genetic diversity, population structure and genome-wide marker-trait association analysis emphasizing seed nutrients of the USDA pea (*Pisum sativum* L.) core collection. *Genes Genomics* 34 305–320. 10.1007/s13258-011-0119-9

[B84] Larsen R. N. Mann N. J. Maclean E. Shaw J. E. (2011). The effect of high-protein, low-carbohydrate diets in the treatment of type 2 diabetes: a 12 month randomised controlled trial. *Diabetologia* 54 731–740. 10.1007/s00125-011-2098-4 21246185

[B85] Leenders M. Verdijk L. B. van der Hoeven L. Adam J. J. van Kranenburg J. Nilwik R. (2013). Patients With Type 2 diabetes show a greater decline in muscle mass, muscle strength, and functional capacity with aging. *J. Am. Med. Dir. Assoc.* 14 585–592. 10.1016/j.jamda.2013.02.006 23537893

[B86] Leenders M. Verdijk L. B. van der Hoeven L. van Kranenburg J. Hartgens F. Wodzig W. K. W. H. (2011). Prolonged leucine supplementation does not augment muscle mass or affect glycemic control in elderly Type 2 diabetic men. *J.* *Nutr.* 141 1070–1076. 10.3945/jn.111.138495 21525248

[B87] Lehmann U. Robin F. (2007). Slowly digestible starch – its structure and health implications: a review. *Trends Food Sci. Technol.* 18 346–355. 10.1016/j.tifs.2007.02.009

[B88] Lestienne I. Besançon P. Caporiccio B. Lullien-Péllerin V. Tréche S. (2005). Iron and zinc in vitro availability in pearl millet flours (*Pennisetum glaucum*) with varying phytate, tannin, and fiber contents. *J. Agric. Food Chem.* 53 3240–3247. 15826084 10.1021/jf0480593

[B89] Lindstrom J. Ilanne-Parikka P. Peltonen M. Aunola S. Eriksson J. G. Hemio K. (2006). Sustained reduction in the incidence of type 2 diabetes by lifestyle intervention: follow-up of the finnish diabetes prevention study. *Lancet* 368 1673–1679. 17098085 10.1016/S0140-6736(06)69701-8

[B90] Liu Q. Donner E. Yin Y. Huang R. L. Fan M. Z. (2006). The physicochemical properties and in vitro digestibility of selected cereals, tubers and legumes grown in China. *Food Chem.* 99 470–477. 10.1016/j.foodchem.2005.08.008

[B91] Ma L. Oei L. Jiang L. Estrada K. Chen H. Wang Z. (2012). Association between bone mineral density and type 2 diabetes mellitus: a meta-analysis of observational studies. *Eur.* *J. Epidemiol.* 27 319–332. 10.1007/s10654-012-9674-x PMC3374119 22451239

[B92] Maiti R. Rodriguez H. G. Kumari C. A. Sarkar N. C. (2016). Macro and micro-nutrient contents of 18 medicinal plants used traditionally to alleviate diabetes in Nuevo Leon, northease of Mexico. *Pak. J. Bot.* 48 271–276.

[B93] Manders R. J. Koopman R. Sluijsmans W. E. van den Berg R. Verbeek K. Saris W. H. (2006). Co-ingestion of a protein hydrolysate with or without additional leucine effectively reduces postprandial blood glucose excursions in Type 2 diabetic men. *J.* *Nutr.* 136 1294–1299. 10.1093/jn/136.5.1294 16614419

[B94] Martin B. C. Warram J. H. Krolewski A. S. Soeldner J. S. Kahn C. R. Martin B. C. (1992). Originally published as Volume 2 Issue 8825Role of glucose and insulin resistance in development of type 2 diabetes mellitus: results of a 25-year follow-up study. *Lancet* 340 925–929. 10.1016/0140-6736(92)92814-V 1357346

[B95] Marz W. Genser B. Drechsler C. Krane V. Grammer T. B. Ritz E. (2011). Atorvastatin and low-density lipoprotein cholesterol in type 2 diabetes mellitus patients on hemodialysis. *Clin. J. Am. Soc. Nephrol.* 6 1316–1325. 10.2215/CJN.09121010 21493741 PMC3109927

[B96] McDougall G. J. Shpiro F. Dobson P. Smith P. Blake A. Stewart D. (2005). different polyphenolic components of soft fruits inhibit α-Amylase and α-Glucosidase. *J.* *Agric. Food Chem.* 53 2760–2766. 10.1021/jf0512095 15796622

[B97] Melnik B. C. (2012). Leucine signaling in the pathogenesis of type 2 diabetes and obesity. *World J. Diab.* 3 38–53. 10.4239/wjd.v3.i3.38 PMC3310004 22442749

[B98] Miedaner T. Korzun V. (2012). Marker-assisted selection for disease resistance in wheat and barley breeding. *Phytopathology* 102 560–566. 10.1094/PHYTO-05-11-0157 22568813

[B99] Mishra S. Monro J. Hedderley D. (2008). Effect of processing on slowly digestible starch and resistant starch in potato. *Starch* 60 8 10.1002/star.200700642

[B100] Møller N. Nair K. S. (2008). Diabetes and protein metabolism. *Diabetes Metab.* *Res. Rev.* 57 3–4. 10.2337/db07-1581 18165354

[B101] Mooradian A. D. Failla M. Hoogwerf B. Maryniuk M. Wylie-Rosett J. (1994). Selected vitamins and minerals in diabetes. *Diab. Care* 17 464–479. 10.2337/diacare.17.5.464 8062625

[B102] Muthamilarasana M. Dhakaa A. Yadav R. Prasad M. (2016). Exploration of millet models for developing nutrient rich graminaceous crops. *Plant Sci.* 242 89–97. 10.1016/j.plantsci.2015.08.023 26566827

[B103] Nambiar V. S. Patwardhan T. (2014). Millets in diabetes - emic views. *Int. J. Pure Appl. Biosci.* 2 89–97.

[B104] Nature Editorial (2012). Misplaced protest. *Nature* 485 147–148. 10.1038/485147b 22575919

[B105] NHS choice (2014). *Type 2 Diabetes.* Available at: http://www.nhs.uk/conditions/Diabetes-type2/Pages/Introduction.aspx

[B106] NHS choice (2015). *Hypoglycaemia (Low Blood Sugar) - Treatment.* Available at: http://www.nhs.uk/Conditions/Hypoglycaemia/Pages/Treatment.aspx

[B107] NIH Osteoporosis and Related Bone Disease National Resource Center (2015). *What People with Diabetes Need to Know About Osteoporosis.* Available at: http://www.niams.nih.gov/health_info/bone/Osteoporosis/Conditions_Behaviors/diabetes.asp

[B108] Nishizawa N. Togawa T. Park K. O. Sato D. Miyakoshi Y. Inagaki K. (2009). Dietary Japanese millet protein ameliorates plasma levels of adiponectin, glucose, and lipids in type 2 diabetic mice. *Biosci.* *Biotechnol. Biochem.* 73 351–360. 10.1271/bbb.80589 19202295

[B109] Norton L. E. Wilson G. J. Layman D. K. Moulton C. J. Garlick P. J. (2012). Leucine content of dietary proteins is a determinant of postprandial skeletal muscle protein synthesis in adult rats. *Nutr. Metab.* 9 1–9. 10.1186/1743-7075-9-67 PMC3488566 22818257

[B110] O’Connell B. S. (2001). Select vitamins and minerals in the management of diabetes. *Diab. Spect.* 14 133–148. 10.2337/diaspect.14.3.133

[B111] Oei L. Rivadeneira F. Zillikens M. C. Oei E. H. G. (2015). Diabetes, diabetic complications, and fracture risk. *Curr. Osteoporosis Rep.* 13 106–115. 10.1007/s11914-015-0260-5 PMC4352609 25648962

[B112] Oei L. Zillikens M. C. Dehghan A. Buitendijk G. H. S. Castaño-Betancourt M. C. Estrada K. (2013). High bone mineral density and fracture risk in Type 2 diabetes as skeletal complications of inadequate glucose control: the rotterdam study. *Diabetes Care* 36 1619–1628. 10.2337/dc12-1188 23315602 PMC3661786

[B113] Pal R. S. Bhartiya A. ArunKumar R. Kant L. Aditya J. P. Bisht J. K. (2016). Impact of dehulling and germination on nutrients, antinutrients, and antioxidant properties in horsegram. *J. Food Sci. Technol.* 53 337–347. 10.1007/s13197-015-2037-3 26787953 PMC4711462

[B114] Paolisso G. D’Amore A. Galzerano D. Balbi V. Giugliano D. Varricchio M. (1993). Daily vitamin E supplements improve metabolic control but not insulin secretion in elderly type II diabetic patients. *Diabetes Care* 16 1433–1437. 8299431 10.2337/diacare.16.11.1433

[B115] Park K.-O. Ito Y. Nagasawa T. Choi M.-R. Nishizawa N. (2008). Effects of dietary korean proso-millet protein on plasma adiponectin, HDL cholesterol, insulin levels, and gene expression in obese Type 2 diabetic mice. *Biosci. Biotechnol. Biochem.* 72 2918–2925. 10.1271/bbb.80205 18997420

[B116] Pawar V. D. Parlikar G. S. (1990). Reducing the polyphenols and phytate and improving the protein quality of pearl millet by dehulling and soaking. *J. Food Sci. Technol.* 27 140–143.

[B117] Pérez-Massot E. Banakar R. Gómez-Galera S. Zorrilla-López U. Sanahuja G. Arjó G. (2012). The contribution of transgenic plants to better health through improved nutrition: opportunities and constraints. *Genes Nutr.* 8 29–41. 10.1007/s12263-012-0315-5 22926437 PMC3534993

[B118] Pflipsen M. C. Oh R. C. Saguil A. Seehusen D. A. Seaquist D. Topolski R. (2009). The prevalence of vitamin B(12) deficiency in patients with type 2 diabetes: a cross-sectional study. *J. Am. Board Fam. Med.* 22 528–534. 10.3122/jabfm.2009.05.090044 19734399

[B119] Pilate G. Guiney E. Holt K. Petit-Conil M. Lapierre C. Leple J.-C. (2002). Field and pulping performances of transgenic trees with altered lignification. *Nat. Biotechnol.* 20 607–612. 10.1038/nbt0602-607 12042866

[B120] Pittas A. G. Dawson-Hughes B. Li T. Van Dam R. M. Willett W. C. Manson J. E. (2006). Vitamin D and calcium intake in relation to Type 2 diabetes in women. *Diab. Care* 29 650–656. 10.2337/diacare.29.03.06.dc05-1961 16505521

[B121] Pittas A. G. Lau J. Hu F. B. Dawson-Hughes B. (2007). The Role of Vitamin D and calcium in type 2 Diabetes. A systematic review and meta-analysis. *J. Clin. Endocrinol. Metabo.* 92 2017–2029. 10.1210/jc.2007-0298 PMC2085234 17389701

[B122] Pushparaj F. Urooj A. (2011). Influence of processing on dietary fiber, tannin and in vitro protein digestibility of pearl millet. *Food Nutr. Sci.* 2 895–900. 10.4236/fns.2011.28122

[B123] Ravi S. B. (2004). Neglected millets that save the poor from starvation. *LEISA India* 6 34–36.

[B124] Rehman Z.-U. Shah W. H. (2005). Thermal heat processing effects on antinutrients, protein and starch digestibility of food legumes. *Food Chem.* 91 327–331. 10.1016/j.foodchem.2004.06.019

[B125] Richards R. A. Rebetzke G. J. Watt M. Condon A. G. Spielmeyer W. Dolferus R. (2010). Breeding for improved water productivity in temperate cereals: phenotyping, quantitative trait loci, markers and the selection environment. *Funct. Plant Biol.* 37 85–97. 10.1071/FP09219

[B126] Roopa S. Premavalli K. S. (2008). Effect of processing on starch fractions in different varieties of finger millet. *Food Chem.* 106 875–882. 10.1016/j.foodchem.2006.08.035

[B127] Rubin M. R. (2015). Bone cells and bone turnover in diabetes mellitus. *Curr. Osteopor. Rep.* 13 186–191. 10.1007/s11914-015-0265-0 25740570

[B128] Saito M. Fujii K. Soshi S. Tanaka T. (2006). Reductions in degree of mineralization and enzymatic collagen cross-links and increases in glycation-induced pentosidine in the femoral neck cortex in cases of femoral neck fracture. *Osteoporos. Int.* 17 986–995. 16552468 10.1007/s00198-006-0087-0

[B129] Sajilata M. G. Singhal R. S. Kulkarni P. R. (2006). Resistant Starch–A review. *Compr. Rev. Food Sci. Food Safety* 5 1–17. 10.1111/j.1541-4337.2006.tb00076.x 33412740

[B130] Salas-Salvado J. Martinez-Gonzalez M. A. Bullo M. Ros E. (2011). The role of diet in the prevention of type 2 diabetes. *Nutr.* *Metab. Cardiovasc. Dis.* 21(Suppl. 2) B32–B48. 10.1016/j.numecd.2011.03.009 21745730

[B131] Sample I. (2012). *Anti-GM Activists Urged Not to Trash Wheat Field.* Available at: http://www.theguardian.com/uk/2012/may/01/anti-gm-activists-wheat-rothamsted?newsfeed=true

[B132] Serraj R. Hash C. T. Rizvi S. M. H. Sharma A. Yadav R. S. Bidinger F. R. (2005). Recent advances in marker-assisted selection for drought tolerance in pearl millet. *Plant Product. Sci.* 8 334–337. 10.1626/pps.8.334

[B133] Shahidi F. Chandrasekara A. (2013). Millet grain phenolics and their role in disease risk reduction and health promotion: a review. *J. Funct. Foods* 5 570–581. 10.1016/j.jff.2013.02.004

[B134] Shao Y. Jin L. Zhang G. Lu Y. Shen Y. Bao J. (2010). Association mapping of grain color, phenolic content, flavonoid content and antioxidant capacity in dehulled rice. *Theor. Appl. Genet.* 122 1005–1016. 10.1007/s00122-010-1505-4 21161500

[B135] Sharma P. C. Singh D. Sehgal D. Singh G. Hash C. T. Yadav R. S. (2014). Further evidence that a terminal drought tolerance QTL is also associated with reduced salt uptake. *Environ. Exp. Bot.* 102 48–57. 10.1016/j.envexpbot.2014.01.013 24895469 PMC4003388

[B136] Sharma S. Agrawal R. P. Choudhary M. Jain S. Goyal S. Agarwal V. (2011). Beneficial effect of chromium supplementation on glucose, HbA1C and lipid variables in individuals with newly onset type-2 diabetes. *J. Trace Elem. Med. Biol.* 25 149–153. 10.1016/j.jtemb.2011.03.003 21570271

[B137] Shobana S. Harsha M. R. Platel K. Srinivasan K. Malleshi N. G. (2010). Amelioration of hyperglycaemia and its associated complications by finger millet (*Eleusine coracana* L.) seed coat matter in streptozotocin-induced diabetic rats. *Br. J. Nutr.* 104 1787–1795. 10.1017/S0007114510002977 20979682

[B138] Singh J. Dartois A. Kaur L. (2010). Starch digestibility in food matrix: a review. *Trends Food Sci. Technol.* 21 168–180. 10.1016/j.tifs.2009.12.001

[B139] Slavin J. (2003). Impact of the proposed definition of dietary fiber on nutrient databases. *J. Food Composition Anal.* 16 287–291. 10.1016/S0889-1575(03)00053-X

[B140] Slavin J. L. (2005). Dietary fiber and body weight. *Nutrition* 21 411–418. 10.1016/j.nut.2004.08.018 15797686

[B141] Sluijs I. (2011). *Diet, Intermediate Risk Markers and Risk of Type 2 Diabetes.* Ph.D. Julius Center, Utrecht University, UMC Utrecht.

[B142] Sluijs I. van der Schouw Y. T. van der A. D. Spijkerman A. M. Hu F. B. Grobbee D. E. (2010). Carbohydrate quantity and quality and risk of type 2 diabetes in the European prospective investigation into cancer and nutrition-netherlands (EPIC-NL) study. *Am.* *J. Clin. Nutr.* 92 905–911. 10.3945/ajcn.2010.29620 20685945

[B143] Snow P. O’Dea K. (1981). Factors affecting the rate of hydrolysis of starch in food. *Am.* *J. Clin. Nutr.* 34 2721–2727. 10.1093/ajcn/34.12.2721 6172034

[B144] Sone H. Tanaka S. Tanaka S. Iimuro S. Oida K. Yamasaki Y. (2011). Serum level of triglycerides is a potent risk factor comparable to LDL cholesterol for coronary heart disease in japanese patients with type 2 diabetes: subanalysis of the japan diabetes complications study (JDCS). *J. Clin. Endocrinol. Metab.* 96 3448–3456. 10.1210/jc.2011-0622 21865372

[B145] Strotmeyer E. S. (2013). *Diabetes and Bone Health.* Available at: http://www.diabetesselfmanagement.com/about-diabetes/general-diabetes-information/diabetes-and-bone-health/18630371

[B146] Suma P. F. Urooj A. (2012). Antioxidant activity of extracts from foxtail millet (*Setaria italica*). *J. Food Sci. Technol.* 49 500–504. 10.1007/s13197-011-0300-9 23904660 PMC3550894

[B147] Suma P. F. Urooj A. (2014). Influence of germination on bioaccessible iron and calcium in pearl millet (*Pennisetum typhoideum*). *J. Food Sci. Technol.* 51 976–981. 10.1007/s13197-011-0541-7 24803707 PMC4008749

[B148] The Look Ahead Research Group (2010). Long term effects of a lifestyle intervention on weight and cardiovascular risk factors in individuals with Type 2 diabetes: four year results of the look AHEAD trial. *Arch.* *Intern. Med.* 170 1566–1575. 10.1001/archinternmed.2010.334 PMC3084497 20876408

[B149] The Telegraph (2015). *‘Pointless’ €3m GM Wheat Trial Fails.* Available at: http://www.telegraph.co.uk/news/earth/agriculture/geneticmodification/11698763/Pointless-3m-GM-wheat-trial-fails.html

[B150] Thornalley P. J. Rabbani N. (2010). Therapy: vitamin B6 B9 and B12 in diabetic nephropathy–beware. *Nat. Rev. Endocrinol.* 6 477–478. 10.1038/nrendo.2010.124 20720587

[B151] Thorne M. J. Thompson L. U. Jenkins D. J. (1983). Factors affecting starch digestibility and the glycemic response with special reference to legumes. *Am.* *J. Clin. Nutr.* 38 481–488. 10.1093/ajcn/38.3.481 6310984

[B152] Thudi M. Gaur P. M. Krishnamurthy L. Mir R. R. Kudapa H. Fikre A. (2014). Genomics-assisted breeding for drought tolerance in chickpea. *Funct. Plant Biol.* 41 1178–1190. 10.1071/FP13318 32481067

[B153] Torres A. M. Avila C. M. Gutierrez N. Palomino C. Moreno M. T. Cubero J. I. (2010). Marker-assisted selection in faba bean (*Vicia faba* L.). *Field Crops Res.* 115 243–252. 10.1016/j.fcr.2008.12.002

[B154] Trichopoulou A. Costacou T. Bamia C. Trichopoulos D. (2003). Adherence to a mediterranean diet and survival in a greek population. *New Engl. J. Med.* 348 2599–2608. 10.1056/NEJMoa025039 12826634

[B155] Trichopoulou A. Kouris-Blazos A. Wahlqvist M. L. Gnardellis C. Lagiou P. Polychronopoulos E. (1995). Diet and overall survival in elderly people. *BMJ* 311 1457–1460. 10.1136/bmj.311.7018.1457 8520331 PMC2543726

[B156] Vallance J. H. Wilson P. J. Leese G. P. McAlpine R. MacEwen C. J. Ellis J. D. (2008). Diabetic retinopathy: more patients, less laser: a longitudinal population-based study in Tayside, *Scotland. Diab. Care* 31 1126–1131. 10.2337/dc07-1498 18346993

[B157] Varshney R. K. Song C. Saxena R. K. Azam S. Yu S. Sharpe A. G. (2013). Draft genome sequence of chickpea (*Cicer arietinum*) provides a resource for trait improvement. *Nat. Biotechnol.* 31 240–246. 10.1038/nbt.2491 23354103

[B158] Varshney R. K. Thudi M. Nayak S. N. Gaur P. M. Kashiwagi J. Krishnamurthy L. (2014). Genetic dissection of drought tolerance in chickpea (*Cicer arietinum* L.). *Theor. Appl. Genet.* 127 445–462. 10.1007/s00122-013-2230-6 24326458 PMC3910274

[B159] Vestergaard P. (2006). Discrepancies in bone mineral density and fracture risk in patients with type 1 and type 2 diabetes—a meta-analysis. *Osteoporos. Int.* 18 427–444. 17068657 10.1007/s00198-006-0253-4

[B160] Vincent J. B. (2007). *The Nutritional Biochemistry of Chromium (III).* Amsterdam: Elsevier.

[B161] Weyer C. Tataranni P. A. Bogardus C. Pratley R. E. (2001). Insulin resistance and insulin secretory dysfunction are independent predictors of worsening of glucose tolerance during each stage of Type 2 diabetes development. *Diabetes Care* 24 89–94. 10.2337/diacare.24.1.89 11194248

[B162] WHO (2016). *Global Report on Diabetes.* Geneva: WHO.

[B163] Willett W. C. Sacks F. Trichopoulou A. Drescher G. Ferro-Luzzi A. Helsing E. (1995). Mediterranean diet pyramid: a cultural model for healthy eating. *Am. J. Clin. Nutr.* 61 1402S–1406S. 7754995 10.1093/ajcn/61.6.1402S

[B164] Wing R. R. Lang W. Wadden T. A. Safford M. Knowler W. C. Bertoni A. G. (2011). Benefits of modest weight loss in improving cardiovascular risk factors in overweight and obese individuals with Type 2 diabetes. *Diabetes Care* 34 1481–1486. 21593294 10.2337/dc10-2415 PMC3120182

[B165] Wong J. H. Lau T. Cai N. Singh J. Pedersen J. F. Vensel W. H. (2009). Digestibility of protein and starch from sorghum (Sorghum bicolor) is linked to biochemical and structural features of grain endosperm. *J. Cereal Sci.* 49 73–82. 10.1016/j.jcs.2008.07.013

[B166] Wood P. J. Braaten J. T. Scott F. W. Riedel D. Poste L. M. (1990). Comparisons of viscous properties of oat and guar gum and the effects of these and oat bran on glycemic index. *J.* *Agric. Food Chem.* 38 753–757. 10.1021/jf00093a036

[B167] Yadav R. S. Howarth C. J. Hash C. T. Witcombe J. R. Khairwal I. S. (2013). “Successful marker-assisted selectionfor disease resistance and drought tolerance in pearl millet in India,” in *Biotechnologies at Work for Smallholders: Case Studies from Developing Countries in Crops, Livestock and Fish* eds Ruane J. Dargie J. Mba C. Boettcher P. Makkar H. Bartley D. (Rome: Food and Agriculture Organization) 18–26.

[B168] Yadav R. S. Sehgal D. Vadez V. (2011). Using genetic mapping and genomics approaches in understanding and improving drought tolerance in pearl millet. *J.* *Exp. Bot.* 62 397–408. 10.1093/jxb/erq265 20819788

[B169] Yin R. V. Phung O. J. (2015). Effect of chromium supplementation on glycated hemoglobin and fasting plasma glucose in patients with diabetes mellitus. *Nutr. J.* 14 1–9. 10.1186/1475-2891-14-14 25971249 PMC4430034

[B170] Zhang G. Ao Z. Hamaker B. R. (2006a). Slow digestion property of native cereal starches. *Biomacromolecules* 7 3252–3258. 10.1021/bm060343a 17096558

[B171] Zhang G. Venkatachalam M. Hamaker B. R. (2006b). Structural basis for the slow digestion property of native cereal starches. *Biomacromolecules* 7 3259–3266. 10.1021/bm050840b 17096559

[B172] Zhang G. Hamaker B. R. (2009). Slowly digestible starch: concept, mechanism, and proposed extended glycemic index. *Crit.* *Rev. Food Sci. Nutr.* 49 852–867. 10.1080/10408390903372466 19960393

[B173] Zhang J. Wang Z.-W. Shi X.-M. (2009). Effect of microwave heat/moisture treatment on physicochemical properties of Canna edulis Ker starch. *J. Sci. Food Agric.* 89 653–664. 10.1002/jsfa.3497

[B174] Zhang X. Zhang G. Guo L. Wang H. Zeng D. Dong G. (2011). Identification of quantitative trait loci for Cd and Zn concentrations of brown rice grown in Cd-polluted soils. *Euphytica* 180 173–179. 10.1007/s10681-011-0346-9

[B175] Zhang Y. Guo K. LeBlanc R. E. Loh D. Schwartz G. J. Yu Y. H. (2007). Increasing dietary leucine intake reduces diet-induced obesity and improves glucose and cholesterol metabolism in mice via multimechanisms. *Diabetes* 56 1647–1654. 10.2337/db07-0123 17360978

